# Metastatic Malignant Melanoma Masquerading as Tolosa–Hunt Syndrome

**DOI:** 10.1155/crop/9124926

**Published:** 2026-07-10

**Authors:** Bashaer Aldhahwani, Benjamin I. Meyer, Rodrigo Santoscoy-Valencia, Joshua Pasol

**Affiliations:** ^1^ Department of Ophthalmology, Bascom Palmer Eye Institute, University of Miami Miller School of Medicine, Miami, Florida, USA, miami.edu; ^2^ Department of Ophthalmology, King Abdulaziz Medical City-National Guard Hospital, Jeddah, Saudi Arabia; ^3^ College of Medicine, King Saud Bin Abdulaziz, University for Health Sciences, Jeddah, Saudi Arabia, medcol.mw; ^4^ Department of Pathology and Laboratory Medicine, University of Miami/Jackson Memorial Hospital, Miami, Florida, USA, miami.edu

**Keywords:** cavernous sinus, Tolosa–Hunt syndrome, uveal melanoma

## Abstract

**Background:**

This case report discusses cavernous sinus metastases arising from uveal melanoma, which manifested after a 7‐year remission period. The diagnosis of systemic metastatic disease was made based on ocular symptoms. To our knowledge, this is the second reported case of metastatic uveal melanoma to the cavernous sinus. The initial presentation posed challenges as it resembled Tolosa–Hunt syndrome, supported by neuroimaging and response to steroid treatment. Initially, suspicion of malignancy was low due to the 7‐year remission period with no ocular or systemic signs suggestive of metastasis. However, repeat imaging was performed due to worsening symptoms upon tapering of steroid treatment. Subsequently, a biopsy was obtained which confirmed the diagnosis.

**Case Presentation:**

A 76‐year‐old male with a history of left eye choroidal melanoma treated 7 years earlier and achieving complete remission on subsequent follow‐ups presented with worsening pain, diplopia, and left ophthalmoplegia upon tapering steroids. An initial MRI suggested Tolosa–Hunt syndrome. A follow‐up MRI revealed enlargement of the left cavernous sinus with enhancing soft tissue, extending into the orbital fissures and foramen rotundum. A left orbital‐zygomatic craniotomy biopsy confirmed malignant melanoma metastasis originating from the choroidal melanoma.

**Conclusion:**

This case underscores the challenge of overlapping clinical presentations and the potential for delayed recognition of metastatic malignancies in unusual sites. Therefore, it emphasizes the importance of vigilant follow‐up and consideration of surgical biopsy, if feasible, to explore alternative causes

## 1. Background

Tolosa–Hunt syndrome (THS) is an idiopathic granulomatous inflammatory disease that affects the cavernous sinus, superior orbital fissure, or orbit, and typically causes ocular pain and ophthalmoplegia [1]. It is a diagnosis of exclusion, as other serious, life‐threatening pathologies may manifest clinically and radiographically similarly [1]. Here, we present a rare case of metastatic melanoma involving the left cavernous sinus which masqueraded initially as THS.

## 2. Case Presentation

A 76‐year‐old male presented with 1 week of left‐sided headaches, periorbital pain, binocular diplopia, and complete left upper eyelid ptosis. Medical history included right middle cerebral artery stroke, atrial fibrillation, coronary artery disease postbypass graft surgery, hypertension, diabetes, hyperlipidemia, and benign prostatic hypertrophy.

He had a remote history of left eye choroidal melanoma treated 7 years prior with Iodine‐125 episcleral plaque radiotherapy and adjuvant crizotinib for 1 year, with complete remission on all subsequent follow‐ups. Four years prior to presentation, he developed radiation‐induced cataract and neovascular glaucoma in the left eye, treated with cataract extraction, panretinal photocoagulation (PRP), and intravitreal anti‐VEGF therapy with eventual quiescence. His best corrected visual acuity in the left eye was resultantly 20/800.

On presentation to the neuro‐ophthalmology clinic, visual acuity was 20/20 in the right eye and hand motion only in the left eye. He had a relative afferent pupillary defect and a middilated pupil in the left eye. Intraocular pressures were normal. Slit‐lamp examination and dilated fundus examination were normal in the right eye and demonstrated laser scars from PRP in the left eye, without recurrent choroidal lesions or neovascularization. Extraocular motility demonstrated complete ophthalmoplegia of the left eye. He endorsed left‐sided V1 hypoesthesia.

MRI brain and orbit with and without contrast revealed enhancement of the left cavernous sinus, extending from the left orbital apex to the inferior orbital fissure (Figure 1A,B), concerning THS. The patient was admitted and treated with 5 days of intravenous methylprednisolone, which initially improved the patient’s pain and dysmotility. A lumbar puncture was performed, revealing an opening pressure of 15 mm H_2_O. Cerebrospinal fluid (CSF) analysis demonstrated a white blood cell count of 2 cells/*μ*L, a red blood cell count of 0 cells/*μ*L, a glucose level of 70 mg/dL (simultaneous serum glucose: 123 mg/dL), and an elevated protein level of 55 mg/dL. Cytological evaluation of the CSF was negative for malignant cells, and the meningoencephalitis panel, cultures, myelin basic protein, oligoclonal bands, IgG, flow cytometry, and paraneoplastic markers were all negative.

**Figure 1:**
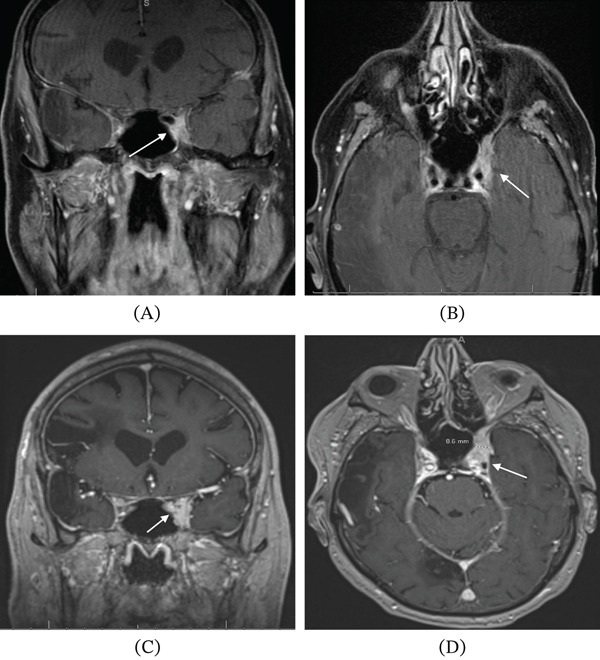
MRI of the orbit and brain with contrast: (A) coronal and (B) axial view shows asymmetry of the cavernous sinuses with prominence on the left. Enhancement extends to the left orbital apex with suspected impingement of the cisternal optic nerve. MRI brain with contrast: (C) coronal and (D) axial view shows worsening asymmetric prominence and enhancement of the left cavernous sinus extending to the left orbital apex, inferior and superior orbital fissures, and left foramen rotundum.

The patient was discharged on a prolonged oral steroid taper. Two months following hospitalization, his pain and diplopia began worsening. Repeat MRI (10 weeks from presentation) showed an interval enlargement of the enhancing lesion in the left cavernous sinus, extending to the orbital fissures and foramen rotundum (Figure 1C,D). Consequently, the patient underwent a left orbito‐zygomatic craniotomy for biopsy, with final pathology indicating malignant melanoma (Figure 2A–C). The patient was diagnosed with choroidal melanoma metastatic to the left cavernous sinus. He completed 37.5 Gy/15 fractions stereotactic radiotherapy to the left cavernous sinus. Serial brain imaging and whole‐body PET/CT scans showed a 1.5 cm left lateral hepatic lesion compatible with metastatic disease.

**Figure 2:**
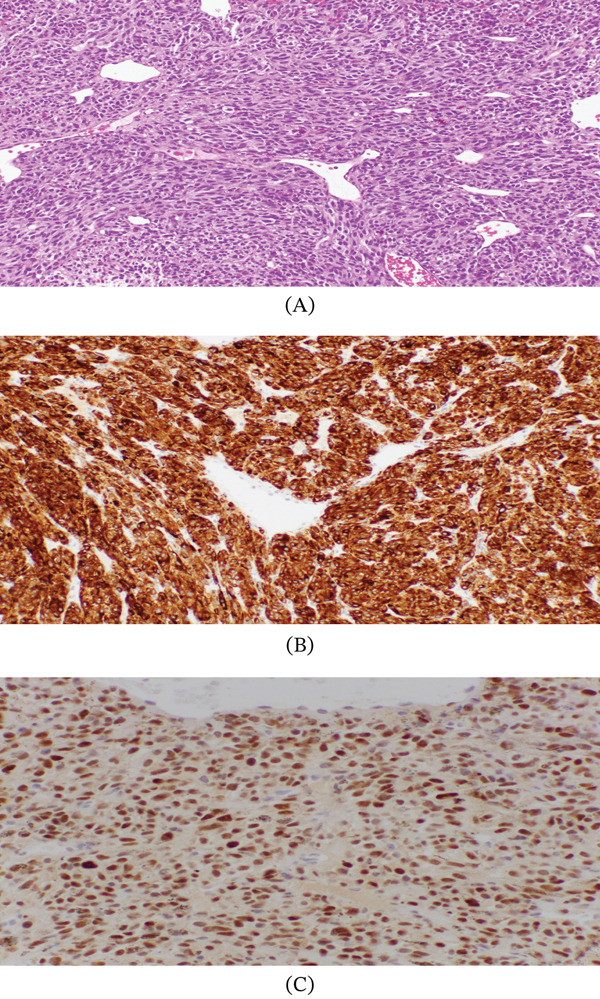
(A) A high‐power view of a malignant melanoma shows a markedly atypical proliferation of epithelioid‐shaped cells with numerous mitoses. (B) Immunohistochemistry for panmelanocyte cocktail is positive in the melanoma cells, staining the cytoplasm of the tumor cells. (C) Immunohistochemistry for PRAME shows positive > 75% of tumor cells show expression, intensity 4+ staining the nuclei of the tumor cells.

## 3. Discussion

Melanoma of the cavernous sinus is exceptionally rare, whether primary or metastatic. To our knowledge, this is only the second reported case of metastatic uveal melanoma to the cavernous sinus. The only previously reported case presented with intermittent diplopia and headache, and neuroimaging was suggestive of a meningioma which prompted an excisional biopsy confirming melanoma. [2] Our case presented with symptoms and neuroimaging suggestive of THS, and initially improved with intravenous and oral steroids.

This case highlights the importance of a thorough investigation of cavernous sinus lesions, for which the differential is broad [3]. When evaluating painful ophthalmoplegia localized to the cavernous sinus, clinicians must navigate a complex landscape of inflammatory, infectious, vascular, traumatic, or neoplastic etiologies. Although THS represents a primary idiopathic inflammatory disease, other nonneoplastic conditions must be systematically ruled out, including granulomatous disorders (such as sarcoidosis, granulomatosis with polyangiitis, and IgG4‐related disease), fungal infections (such as mucormycosis and aspergillosis), and vascular anomalies like carotid‐cavernous fistulas or intracavernous aneurysms. Clinical manifestations of all of these can include sudden ophthalmoplegia and pain, which may resemble THS. There is a nonzero risk of misdiagnosis if a complete workup is not obtained [4].

Neoplastic invasion represents another critical diagnostic category. Primary tumors of the skull base, such as meningiomas, schwannomas, and chordomas, frequently arise in this region. Conversely, metastatic disease to the cavernous sinus accounts for only a small percentage of skull base malignancies, with primary tumors most commonly originating from breast, lung, and prostate carcinomas [5]. Cutaneous melanoma is well‐known for its neurotropic propensity, but secondary metastasis from a primary uveal melanoma remains an extreme rarity. Secondary involvement typically occurs via hematogenous seeding to the highly vascular cavernous sinus or, less commonly, via retrograde perineural extension along the ciliary nerves through the orbital apex [2]. This clinical manifestation can be the first overt sign of an underlying systemic malignancy [6].

Cavernous sinus melanoma (primary or metastatic) has been reported in only a handful of case reports, summarized in Table 1 [2, 6–9].

**Table 1:** A literature review of histopathologic‐confirmed or radiographic‐presumed cavernous sinus melanoma (primary or metastatic).

Authors	Case description	Histopathology/genetic profile result from the cavernous sinus lesion	Histopathology/genetic profile result from another site	Treatment	Outcome
Assi et al. [2]	50 YO with history of choroidal melanoma treated with brachytherapy. Developed isolated metastasis to the cavernous sinus 7 years later. Clinically presenting with intermittent diplopia and headache.	Many of the tumor cells contained melanin, and the neoplastic cells were intensely immunoreactive using a cocktail of antibodies to the melanoma markers MART‐1 (Melan‐A) and HMB‐45 (Figure 2B), weakly to moderately intense and focally reactive using antibodies to S100 protein, and the tumor cells were nonreactive to a cocktail of antibodies to low‐ and high‐weight cytokeratins. Next‐generation sequencing revealed mutations in *GNA11* (c.626A > T; p.Gln209Leu), but no mutations in *BRAF*, *NRAS*, or *KIT* were detected.	None	5 sessions of 5 Gy hypofractionated stereotactic radiotherapy followed by 27 Gy stereotactic radiosurgery after 20 months due to signs of progression.	The diplopia resolved and serial neurological evaluation showed stability over 6 months.
El‐Kalliny et al. [6]	61 YO with cavernous sinus syndrome in the form of ipsilateral multiple cranial nerve palsies.	Presumed primary malignant melanoma.	None	Surgery (frontoparietal craniotomy for tumor excision).	Tumor recurrence 6 months after the first operation requiring 2nd exploration and whole‐brain radiation.
Garge et al. [7]	36 YO with a left cavernous sinus mass lesion. Clinical presentation of headaches with ipsilateral multiple cranial nerve palsies.	Melanocytic neoplasm with high activity consistent with malignant melanoma.	None	CT‐guided stereotactic radiosurgery using 16 Gy radiation.	Improved symptoms 1 month after radiation at last follow‐up.
Harkness et al. [8]	21 YO with cutaneous melanoma manifesting initially as cavernous sinus syndrome (headaches, diplopia, and multiple cranial nerve palsies) secondary to metastasis.	None	Immunostains showed strong expression of S100 and HMB45 consist with metastatic malignant melanoma.	Whole brain radiotherapy.	The patient developed multiple metastases and died 4 months following initial presentation.
Dryden et al. [9]	57 YO with recurrent cutaneous melanoma—refractory to several excisional and radiation therapies—metastatic to the cavernous sinus and clinically presenting with ipsilateral Horner syndrome and abducens palsy.	None	Malignant melanoma (right forehead).	Nivolumab and ipilimumab.	Last follow up on the 3rd cycle and patient was stable.

Abbreviations: MRI, magnetic resonance imaging; YO, years old.

MRI is critical for detecting cavernous sinus lesions. However, differentiating an atypical presentation of inflammatory cavernous sinus disease from a metastatic lesion remains a notorious diagnostic challenge [3]. In our case, the patient′s initial presentation strongly mimicked the macroscopic MRI findings of THS, leading to a deferral of early incisional biopsy. Nevertheless, specific neuroradiologic nuances can provide critical retrospective or prospective clues pointing toward a metastatic etiology.

Unlike classic inflammatory lesions, which are typically isointense to hypointense on T1‐weighted sequences, melanotic melanoma metastases often exhibit a distinct intrinsic T1 hyperintensity and T2 hypointensity caused by the paramagnetic properties of melanin or subclinical intratumoral hemorrhage [10]. Furthermore, malignant lesions typically demonstrate restricted diffusion on diffusion‐weighted imaging (DWI) and corresponding apparent diffusion coefficient (ADC) maps due to high tumor cell density—a feature crucial for differentiating highly cellular malignant pathologies from classic idiopathic inflammation [11]. Diagnostic attention must also be paid to patterns of contrast enhancement, osseous destruction of the surrounding skull base, and signs of perineural spread along the traversing cranial nerves (CN III, IV, V, VI), which strongly favor melanoma malignancy over a primary, localized inflammatory process [3, 12].

In addition to radiologic ambiguities, the patient′s transient clinical improvement following corticosteroid administration heavily contributed to the initial diagnostic confusion. A rapid response to systemic steroids is considered a hallmark feature of THS and is frequently utilized as a de facto diagnostic criterion. However, temporary symptomatic relief is not pathognomonic for benign inflammation. [13]

In neoplastic conditions such as metastatic uveal melanoma, corticosteroids can induce a dramatic, albeit transient, regression of symptoms through two primary mechanisms. First, steroids stabilize tumor‐compromised capillary membranes, rapidly decreasing vasogenic peritumoral edema and reducing the mass effect within the rigid confines of the cavernous sinus [14]. Second, they aggressively suppress the secondary localized inflammatory cascade and cytokine release incited by the tumor cells themselves. This secondary inflammatory suppression can even lead to a transient decrease in the size of the cavernous sinus lesion on follow‐up neuroimaging, closely mimicking the structural resolution of a primary inflammatory process [15]. Clinicians must therefore remain cognizant that steroid responsiveness can create a “diagnostic honeymoon period,” masquerading as THS and dangerously delaying tissue confirmation in patients with an oncologic history.

The decision to perform a surgical biopsy early on should be based on an index of suspicion for alternative etiologies, possibly manifesting as atypical symptoms, MRI findings, or medical history, combined with the ease of obtaining a tissue specimen. In the only other reported case of uveal melanoma with cavernous sinus metastasis, the lesion was felt to be a meningioma, which is what ultimately prompted tissue diagnosis [2].

In our case, the cavernous sinus was the first sign of metastasis 7 years after diagnosis and treatment of the primary uveal melanoma. The risk of uveal melanoma metastasis depends on several factors, including tumor morphology, histopathology, location, and genetic expression. Recently, gene expression profiles are proving more precise than other prognostic modalities in predicting the risk of metastasis [16]. This has led to a new classification of tumors based on a 5‐year tumor‐related mortality rate: Class 1A tumors with a low 2%; Class 1B with a higher 21%; and Class 2 with a significant 72% [16]. Our case was categorized as Class 2 based on the gene expression profile. Other emerging molecular markers, such as abnormalities in Chromosomes 3, 6, 8, Monosomy 3, and mutations in the tumor suppressor gene *BAP1*, have shown links to higher risks of metastasis [16].

Our report has certain limitations, primarily dictated by its retrospective nature and constraints in data availability. Detailed cytogenetic findings and specific molecular prognostic markers of the primary ocular tumor—beyond the Class 2 gene expression profile classification—were unavailable. Furthermore, the patient was unfortunately lost to follow‐up shortly after completing palliative radiotherapy. This restricts our ability to document long‐term neurological recovery, evaluate delayed systemic progression, or provide comprehensive survival outcomes. Despite these data constraints, the exceptionally rare clinical presentation and the histopathological confirmation of the cavernous sinus lesion retain significant instructional value for neuro‐ophthalmologists and ocular oncologists.

In summary, painful ophthalmoplegia and cavernous sinus enhancement may be from THS; however, this is a diagnosis of exclusion. In the right clinical context, a surgical biopsy may be necessary for accurate diagnosis. Specifically, in patients with a history of uveal melanoma, one must maintain a high index of suspicion for metastasis or alternative diagnoses, particularly in cases with an unusual response to treatment.

## Funding

No funding was received for this manuscript.

## Disclosure

All authors attest that they meet the current ICMJE criteria for Authorship.

## Consent

Verbal consent was obtained from the patient to prepare and publish this case report.

## Conflicts of Interest

The authors declare no conflicts of interest.

## Data Availability

Data sharing not applicable to this article as no datasets were generated or analyzed during the current study.
